# A ComX-based strategy for artificially inducing competence in naturally non-transformable *Streptococcus parasanguinis*

**DOI:** 10.1128/aem.00082-26

**Published:** 2026-03-18

**Authors:** Hui-Ru Shieh, Yu-Shan Yen, Yu-Juan Lin, Yi-Ywan M. Chen

**Affiliations:** 1 Department of Microbiology and Immunology, College of Medicine, Chang Gung University 71589, Taoyuan, Taiwan; 2 Graduate Institute of Biomedical Sciences, College of Medicine, Chang Gung University 210836, Taoyuan, Taiwan; 3 Molecular Infectious Disease Research Center, Chang Gung Memorial Hospital Linkou 38014, Linkou, Taiwan; Washington University in St Louis, St. Louis, Missouri, USA

**Keywords:** *Streptococcus salivarius*, *Streptococcus parasanguinis*, competence, σ^X^, natural transformation

## Abstract

Many streptococci, including strains of *Streptococcus salivarius* and *Streptococcus parasanguinis*, harbor *com* genes but do not exhibit natural competence. In *S. salivarius* YS18, a chromosomal rearrangement in the 5′ flanking region of *comX*, encoding σ^X^, the alternative sigma factor responsible for the late *com* gene expression, was found to confer competence, whereas its parental strain 57.I was not competent. This genetic arrangement led to constitutive, high-level *comX* expression, with ComX production independent of quorum-sensing regulation. By establishing YS18 *comX* on the *Streptococcus-Escherichia coli* shuttle vector pDL278 in non-competent *S. parasanguinis* FW213, transient competence could be established, enabling mutant construction via ligation mutagenesis; thereby, bypassing the need to generate plasmids carrying mutated loci that would be introduced via electroporation. Plasmids in the mutant isolates were successfully cured by cultivation in antibiotic-free media post-mutagenesis. In conclusion, the lack of competence in *com* gene-containing streptococci may likely be due to inadequate ComX production. Given the structural conservation of ComX, constitutive *comX* expression provides a practical strategy to establish competence in non-competent streptococcal hosts.

**IMPORTANCE:**

Many streptococci encode *com* genes but remain non-competent, limiting genetic manipulation. This study found that insufficient ComX production was the key barrier to competence development in these strains. However, variations in competence-stimulating peptides and σ^X^-inducing peptides make optimizing *comX* expression with synthetic peptides impractical. Instead, by introducing a constitutively expressed *comX* from *Streptococcus salivarius*, a transient competence stage can be established in non-competent *Streptococcus parasanguinis*, enabling efficient mutant construction via ligation mutagenesis. This approach provides a generalizable strategy to simplify mutagenesis workflows and expands the genetic toolkit for functional studies in otherwise intractable streptococcal strains.

## INTRODUCTION

Natural competence describes the ability of a bacterium to acquire foreign DNA by the process known as transformation (1–3). The machinery for DNA uptake and subsequent integration into host chromosomes in transformation is primarily conserved among bacterial species, except *Helicobacter pylori* (4). Specifically, the type II secretion system, or the type IV (pseudo)pilus system, mediates DNA uptake in most competent bacterial species, whereas *H. pylori* utilizes a type IV secretion system for DNA uptake.

Although the acquisition of exogenous DNA could facilitate DNA repair, enhance genetic diversity, and serve as a nutrient source, the expression of competence is an energetically costly process and may result in deleterious genetic changes (1, 5). Thus, the expression of competence is tightly controlled by various signals in most species (3). In Gram-positive bacteria, the early phase of competence development involves the activation of quorum-sensing (QS) systems, which leads to the expression of a key transcription activator, such as the alternative sigma factor X (σ^X^) of streptococci (6) and the transcription factor ComK of *Bacillus subtilis* (7). The expression of the transcriptional regulator is required to express genes encoding DNA uptake and processing machinery. Based on their roles in competence development, genes encoding QS systems are referred to as early *com* genes, while those involved in DNA uptake and processing are known as late *com* genes.

In streptococci, the expression of *comX*, encoding σ^X^, is activated by two QS systems, ComCDE and ComRS (6, 8). The ComCDE system is mainly found in the Mitis and Anginosus groups (9), whereas the ComRS system, first identified in *Streptococcus thermophilus* and *Streptococcus salivarius* (10), is also present in the Bovis, Mutans, and Pyogenic groups (11, 12) and *Streptococcus suis* (13). Competent streptococci generally utilize either ComCDE or ComRS to regulate *comX* expression, with the exception of *Streptococcus mutans,* in which both systems act synergistically in competence development (14). In the ComCDE system, the *comC*-encoded competence-stimulating peptide (CSP) precursor is processed and exported by ComAB. The extracellular CSP is sensed by the two-component system ComDE, leading to ComD autophosphorylation and subsequent phosphorylation of the response regulator ComE. Phosphorylated ComE binds to a site containing two imperfect repeats separated by 12 bp (DCAYTTBRG-N_12_-ACAKTTSAG) in the promoter regions of early *com* genes (15, 16). This interaction enhances *comAB* and *comCDE* expression through positive feedback (16, 17) and also initiates *comX* transcription (18), thereby triggering competence. In the ComRS system, the σ^X^-inducing peptide (XIP), encoded by *comS*, is processed, exported, and then reimported via an Ami oligopeptide transporter. The intracellular processed XIP activates the regulatory activity of ComR. By binding to a palindromic sequence (TAGTGACAT-N_2_-ATGTCACTA) located in the promoter of *comX* and *comS* (19, 20), ComR-XIP activates *comX* transcription.

The consensus recognition sequence of σ^X^ (TNCGAATA), known as the cin (for competence inducing) box or the ComX box, is commonly located at the 5′ flanking region of late *com* genes (21). Studies in *Streptococcus pneumoniae* further indicated that a T-motif (TTTTT), located 9 bases 5′ to the ComX box, is commonly found in the promoter regions of late *com* genes, confirming the conserved role of ComX in recognizing and activating late *com* gene expression (21, 22). Thus, in contrast to the conservation in the DNA uptake and processing systems, two divergent competence-inducing pathways have evolved among streptococcal species.

*Streptococcus parasanguinis* is an early colonizer and frequent isolate of dental plaque (23). The association between *S. parasanguinis* and life-threatening endocarditis has been observed clinically (24, 25) and analyzed in an animal model (26, 27). Although genes encoding proteins for genetic transformation are observed on the genome of *S. parasanguinis* FW213 (28), the strain is not naturally competent. Consequently, electroporation of plasmids carrying mutated loci isolated from recombinant *Escherichia coli* strains remains the standard approach for generating isogenic mutant strains, limiting functional genetic studies. Here, to overcome this limitation, we developed a competence-inducing system in *S. parasanguinis* FW213 by using the constitutively expressed *comX* from *S. salivarius*. This system enables the generation of mutant strains via ligation mutagenesis (29), a method that bypasses the need to construct mutated loci but requires a relatively high transformation efficiency in the host. With this induced competence, genes previously refractory to cloning in *E. coli* can now be directly mutated in FW213, significantly enhancing its genetic accessibility and experimental utility.

## MATERIALS AND METHODS

### Bacterial strains, plasmids, and culture conditions

Streptococcal strains were incubated routinely at 37°C under 5% CO_2_ in Todd-Hewitt (TH) or brain heart infusion (BHI). To select and maintain recombinant *S. salivarius* strains, erythromycin (Em) at 5 μg mL^−1^, spectinomycin (Sp) at 800 μg mL^−1^, and kanamycin (Km) at 600 μg mL^−1^ were included in the culture medium, as indicated. For recombinant *S. parasanguinis* strains, Em, Sp, and Km were used at 5 μg mL^−1^, 500 μg mL^−1^, and 200 μg mL^−1^, respectively. The *Streptococcus-E. coli* shuttle vectors pDL276 and pDL278 were used to examine transformation efficiency. All strains and plasmids used in this study are listed in Table 1. All primers used in this study are listed in Table S1.

**TABLE 1:** Bacterial strains and plasmids used in this study

Strain or plasmid	Relevant phenotype^*a*^	Description^*b*^	Source
Streptococcal strains			
*S. salivarius* 57.I	CodY^+^, Com^−^	Wild-type *S. salivarius s*train	(30)
*S. salivarius* Δ*codY*	CodY^−^, Com^+^, Em^r^	A *codY-*deletion derivative of 57.I, expressing competence	(31)
*S. salivarius* YS18	Com^+^, CodY^+^, Km^r^	A *comX* expressing strain	This study
*S. salivarius* Δ*amiC*	CodY^−^, Com^+^, Km^r^	Δ*codY amiC*::Ω*kan*	This study
*S. salivarius* Δ*comA*	CodY^−^, Com^+^, Km^r^	Δ*codY comA*::Ω*kan*	This study
*S. salivarius* Δ*comEA*	CodY^−^, Com^+^*, Km^r^	Δ*codY comEA*::Ω*kan*	This study
*S. salivarius* Δ*comFA*	CodY^−^, Com^+^*, Km^r^	Δ*codY comFA*::Ω*kan*	This study
*S. salivarius* Δ*comGA*	CodY^−^, Com^−^, Km^r^	Δ*codY comGA*::Ω*kan*	This study
*S. salivarius* YS5	CodY^−^, Com^+^, Em^r^, Sp^r^	Δ*codY* harboring a *spe-*p*_comGA_-cat* fusion at *lacZ*	This study
*S. salivarius* YS14	CodY^+^, Com^+^, Km^r^, Sp^r^	YS18 harboring a *spe-*p*_comGA_-cat* fusion at *lacZ*	This study
*S. salivarius* YS22	CodY^−^, Com^−^, Em^r^, Sp^r^	Δ*codY* harboring a *spe-*mutated p*_comGA_-cat* fusion at *lacZ*	This study
*S. parasanguinis* FW213	Com^−^	Wild-type *S. parasanguinis* strain	(23)
*S. parasanguinis* FW213/pDL278_*comX*	Sp^r^, Com^+^	FW213 harboring pDL278_*comX*	This study
*S. parasanguinis* Δspaf_1086	Em^r^, Com^−^	FW213 Spaf_1086::*erm*	This study
*S. parasanguinis* Δspaf_1451	Em^r^, Com^−^	FW213 Spaf_1451::*erm*	This study
*S. parasanguinis* Δspaf_1493	Em^r^, Com^−^	FW213 Spaf_1493::*erm*	This study
*S. parasanguinis* Δspaf_2070	Em^r^, Com^−^	FW213 Spaf_2070::*erm*	This study
Plasmids			
pDL276	Km^r^	*Streptococcus-E. coli* shuttle vector with a *Streptococcus* pVA380-1 basic replicon	(32)
pDL278	Sp^r^	*Streptococcus-E. coli* shuttle vector with a *Streptococcus* pVA380-1 basic replicon	(33)
pDL278_*comX*	Sp^r^	pDL278 harboring *comX*	This study
pDot1	Sp^r^	*lacZ* integration vector for *S. salivarius* carrying a *spe-cat* cassette	This study
pYS2	Sp^r^	p*_comGA_* is inserted 5′ to *cat* in pDot1	This study

^*a*^ Com, competence; +, positive; −, negative. Em, erythromycin; Km, kanamycin; Sp, spectinomycin; r, resistance; s, sensitive. *, the transformation efficiency was less than 100 CFU μg^−1^.

^*b*^ Ω*kan*, Km resistance gene flanked by the Ω element; *erm*, Em resistance gene; *spe*, Sp resistance gene; *cat*, chloramphenicol acetyltransferase gene.

### Construction of *S. salivarius* YS18

The *erm-*inactivated *codY* in *S. salivarius* Δ*codY* was replaced with an Ω*kan*-tagged *codY* to generate strain YS18 by ligation mutagenesis (29). Notably, the Ω*kan* cassette (34) contains a *kan* gene flanked by three transcriptional terminators at each end, and its insertion generates a polar mutation. Briefly, a 912 bp fragment containing *alaA* (Ssal_00403) and a 2,191 bp fragment containing intact *codY*, its 5′ flanking region of 254 bp, and the predicted terminator were amplified from *S. salivarius* 57.I using primer pair ala_488_SacI_S + ala_1390_BamHI_AS and codY_815_BamHI_S + codY_3603_AS, respectively. The PCR products were digested with BamHI and then ligated with an Ω*kan-*containing BamHI fragment. The ligation mixture was used to transform *S. salivarius* Δ*codY* (see below), and recombinants were selected for Km resistance. The replacement of *codY::erm* with the Ω*kan*-tagged *codY* locus was confirmed by sequence analysis. The resulting strain YS18 carries a *codY* coding sequence and 5′ flanking region that are identical to those of wild-type 57.I.

### Transformation of competent streptococcal strains

Transformation of competent streptococcal strains was performed using the method of Fontaine et al. (10) with minor modifications. Briefly, overnight cultures were refreshed in BHI at a 1:10 dilution, mixed with 100–300 ng DNA or ligation mixtures, and incubated at 37°C under 5% CO_2_ for 5 h. Transformants were selected on TH or BHI agar containing appropriate antibiotics. The genotype of the antibiotic-resistant transformants was examined by PCR with specific primers. The transformation efficiency was calculated as the number of antibiotic-resistant CFU per μg plasmid DNA used.

### RNA isolation and reverse transcription quantitative real-time PCR

Total cellular RNA was isolated from *S. salivarius* strains grown to log phase (O.D._600_ = 0.6) using the method described by Chen et al*.* (35). DNase I (50 U/μL, Thermo Scientific) was used to remove residual chromosomal DNA. The treated RNA samples were further purified using a Qiagen RNeasy mini kit. Two micrograms of purified RNA was reverse transcribed into cDNA using AMV reverse transcriptase and random primers. Using a KAPA SYBR FAST qPCR Master Mix (2X) Kit in the QuantStudio 3 Real-Time PCR System, expression levels of *comX* in each preparation were determined using the primer pair comX_1025 + comX_1236. Results were analyzed by QuantStudio Design & Analysis Software v1.5.2.

### Purification of recombinant ComX protein, production of polyclonal antisera, and western blot analysis

The coding sequence of *comX* was generated from *S. salivarius* Δ*codY* by PCR using the primer comX_BamHI_S + comX_SphI_AS and cloned into pQE30 (Qiagen) in *E. coli* M15. The identity of the cloned PCR fragment was verified by sequence analysis. His-tagged ComX was expressed and purified under denaturing conditions by Ni-Sepharose affinity chromatography (GE Healthcare) according to the manufacturer’s recommendation. The identity of this recombinant protein was verified by matrix-assisted laser desorption ionization-time of flight mass spectrometry. The concentration of the purified protein was determined using the Bio-Rad Protein Assay based on the method of Bradford (36).

Approximately 2 mg of the purified protein was separated on a 12% SDS-PAGE, and the corresponding protein band was excised from the gel and used to generate polyclonal antiserum in rabbits (Genesis). The specificity and titer of the antiserum were assessed by western blot analysis.

To examine ComX production in streptococcal strains, log phase cultures (O.D._600_ = 0.6) were harvested, washed once with 10 mM Tris, pH 7.8, and resuspended in 1% of the culture volume in the same buffer. Concentrated cell suspensions were subjected to mechanical disruption in the presence of an equal volume of 0.1-mm glass beads by homogenization in a Beadbeater (Biospec Products) for a total of 120 s at 4°C. Fifty micrograms of each sample was analyzed on a 12% SDS-PAGE. For western blot analysis, the separated protein species were transferred to a piece of PVDF membrane. The membrane was blocked overnight at 4°C in PBS containing 0.3% Tween 20 (PBST) and 10% nonfat dry milk. The blot was then incubated for 1 h with anti-ComX antibody diluted 1:2,000 in PBST containing 5% nonfat dry milk. To be noted, when the antiserum was used to detect ComX in *S. parasanguinis* harboring pDL278_*comX*, the antiserum was adsorbed with the total cell lysate of *S. salivarius* 57.I for 1 h at room temperature before use. ComX production was detected using a horseradish peroxidase (HRP)-conjugated goat anti-rabbit secondary antibody (GeneTex) and a luminol-based Immobilon western chemiluminescent HRP substrate (Millipore). Signals were imaged using the e-Blot Touch Imager (e-Blot Life Science).

### Construction of the p*_comGA_-cat* fusion strains

A 215 bp region 5′ to *comGA* was generated from *S. salivarius* 57.I using primers 0141_3465_SmaI_S and GA_3679_SalI_AS, digested with XmaI and SalI, and then ligated with XmaI/SalI-digested plasmid pDot1, a derivative of *S. salivarius* integration vector pMC300 (36), to generate plasmid pYS2 in *E. coli*. Plasmid pDot1 carries a Sp resistance gene (*spe*) (37)-tagged promoterless *cat* located within *lacZ*. The sequence of p*_comGA_* in pYS2 was verified by sequencing. Plasmid pYS2 was introduced into strains Δ*codY* and YS18 by transformation, and the correct integration at the *lacZ* locus in the Sp-resistant (Sp^r^) transformants was confirmed by colony PCR. The resulting strains were designated YS5 and YS14, respectively.

To determine whether ComX was essential for *comGA* expression, the predicted ComX box (TACGAATA) in strain YS5 was replaced with a SmaI recognition sequence (CCCGGG) by ligation mutagenesis. Briefly, two fragments flanking the ComX box were amplified from YS5 using primer pairs lacZ_S + Δbox_GA3662_AS and Δbox_GA3671_SmaI_S + lacZ_AS. The PCR products were phosphorylated with T4 polynucleotide kinase, ligated, and transformed into strain Δ*codY* to generate strain YS22. The integration of a mutated p*_comGA_-cat* region in Sp^r^ transformants in YS22 was amplified with primers lacS_1241 and lacAS_1465 and verified by sequencing.

### Chloramphenicol acetyltransferase assay

Overnight cultures of *S. salivarius* strains were diluted at 1:20 in pre-warmed BHI and grown to O.D._600_ = 0.8. Cells were harvested and washed once with 10 mM Tris-HCl, pH 7.8, and the cell lysate was prepared as described above. The chloramphenicol (Cm) acetyltransferase (CAT) activity of each sample was determined using the CAT assay (38). The activity was calculated as nmole of Cm acetylated min^−1^ mg^−1^ protein. Reactions without Cm are used as the negative control. All measurements were conducted in triplicate.

### 5′ Rapid amplification of cDNA ends

The transcription start site of *comX* in *S. salivarius* YS18 was determined by 5′ rapid amplification of cDNA ends (5′ RACE) (Invitrogen). Total cellular RNA was purified from cultures of YS18 grown to O.D._600_ = 0.6 as described above. Five micrograms of total cellular RNA was used to synthesize *comX-*specific cDNA using primer comX_4470_AS, located 329 bases 3′ to the translation start site of *comX*. The mRNA template was removed using an RNase mix. First-strand cDNA was purified via Snap column, and a poly-C tail was added to the 3′ end of the cDNA using terminal deoxynucleotidyl transferase and dCTP. Tailed cDNA was amplified by PCR using the abridged anchor primer and comX_5143_AS, which is 254 bases 3′ to ATG of *comX*. One percent of the PCR products was again amplified by PCR using the abridged universal amplification primer and comX_5022_AS. The final PCR product was separated by gel electrophoresis and analyzed by sequencing.

### Sequence analysis and protein structure prediction

Protein sequence alignment was analyzed using Muscle, part of the EMBL-EBI suite (https://www.ebi.ac.uk/jdispatcher/psa) (39). The similarity and identity between homologs were analyzed using BlastP from NCBI (https://blast.ncbi.nlm.nih.gov/Blast.cgi). The 3D structures of the ComX proteins were predicted using the AlphaFold protein structure database (https://alphafold.ebi.ac.uk/), accessed on 12 August 2025.

### Establishment of the competence stage in *S. parasanguinis* FW213

A *comX*-containing DNA fragment was amplified from *S. salivarius* YS18 using the primer pair comX_3701_BamHI_S + comX_5029_XhoI_AS. This region includes intact *comX* and its 5′ flanking region of 441 bp. Restriction recognition sequences were included in the primers to facilitate cloning into pDL278. The resulting recombinant plasmid, designated pDL278_*comX*, was initially established in *E. coli* DH10B. Plasmid DNA isolated from the recombinant *E. coli* strain was introduced into *S. parasanguinis* FW213 by electroporation (35) with selection for Sp resistance. The presence of plasmid pDL278_*comX* in the Sp^r^ FW213 isolates was confirmed by PCR with *comX-*specific primers. The competence of *S. parasanguinis* FW213/pDL278_*comX* was investigated using the Km resistance gene (*kan*)-bearing plasmid, pDL276. Transformation was carried out as described above. To be noted, when preparing strain FW213/pDL278_*comX* for genetic transformation, the bacterial cells were initially cultivated in TH containing Sp, and Km was used only to select pDL276-containing isolates post-transformation.

### Assessment of plasmid stability, ComX production, and competence under non-selective conditions

Plasmid stability was examined in the absence of selection as previously described (40). Briefly, FW213 carrying pDL278_*comX* was grown in TH broth containing Sp to ensure plasmid maintenance. The culture was subsequently diluted 10^−6^ into fresh antibiotic-free TH and incubated for 16 h (approximately 20 generations). This serial subculturing was continued for 3 to 4 days. At each daily passage, the transformation efficiency and the ComX level were examined.

### Plasmid curing

For plasmid curing experiments, recombinant mutant strains were cultivated in antibiotic-free TH and passaged as described above. At each passage, the proportion of Sp-sensitive isolates was determined. The absence of *comX*-containing plasmids was confirmed by PCR using *comX-*specific and *spe-*specific primers.

### Construction of isogenic knockout strains in competent streptococcal strains

Ligation mutagenesis was used to generate isogenic mutant strains in competent streptococcal strains (29). Briefly, two DNA fragments, 5′ and 3′ to the target gene, were generated from *S. salivarius* 57.I or *S. parasanguinis* FW213 by PCR with specific primers. The PCR products were restriction digested and then ligated with DNA fragments containing Ω*kan*, or the Em resistance gene (*erm*) (41) in a ligation reaction. The ligation mixture was introduced into competent streptococcal strains (*S. salivarius* Δ*codY*, *S. salivarius* YS18, and *S. parasanguinis* FW213/pDL278_*comX*) via natural transformation. The antibiotic-resistant colonies were selected, and the allelic exchange event in the resistant strains was verified by PCR with specific primers. With *S. parasanguinis* strains, once the allelic exchange event had been confirmed, the *S. parasanguinis* isolates were passaged in plain TH, and the loss of pDL278-*comX* was confirmed by PCR.

## RESULTS

### *comX* expression in *S. salivarius* strains

**Fig 1:**
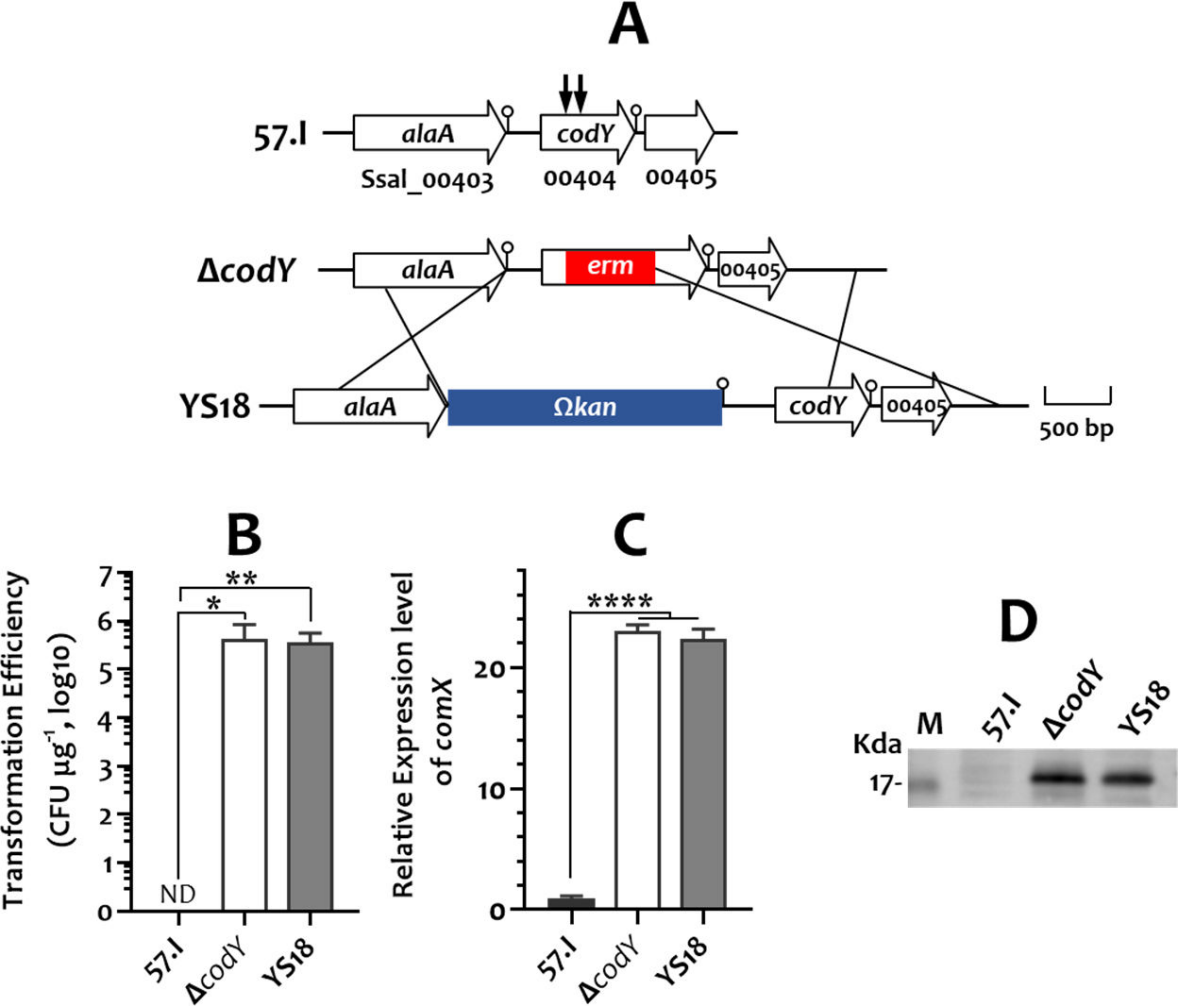
The competence phenotype of *S. salivarius* strains. (**A**) Schematic presentation of *codY* and its flanking loci of *S. salivarius* 57.I, Δ*codY*, and YS18. Gene names and Ssal tag numbers are shown. For uncharacterized loci, only the tag numbers are indicated. The region that was replaced with *erm* in Δ*codY* is indicated by two vertical arrows. A lollipop indicates the predicted transcription terminator. The mutated *codY* was replaced with an Ω*kan-*tagged, wild-type copy of *codY* in YS18. Regions that facilitated allelic exchange are marked with crossed lines. (**B**) Transformation efficiency of *S. salivarius* strains. The transformation efficiency is expressed as CFU μg^−1^ pDL278 in log_10_. ND, not detected. The numbers are the mean and standard deviation of six biological samples. (**C**) The *comX* expression level is quantitated by RT-qPCR. 16S RNA was used as an internal control. The expression level in 57.I was used as a reference. The numbers are the mean and standard deviation of three biological samples. (**D**) Western blot showing ComX protein in *S. salivarius* strains. A 50 µg of total cell lysate was loaded per lane. M, the protein marker. Significant differences between wild-type 57.I and Δ*codY*/YS18 were analyzed using an unpaired Student’s *t*-test. *, *P* < 0.05; **, *P* < 0.01; ****, *P* < 0.0001.

*S. salivarius* 57.I, a urease producer, is not naturally competent. However, in a previous study, we observed competence in a *codY-*deletion mutant (strain Δ*codY*) (31). Since CodY is unknown to participate directly in competence development, it was likely that the competence was due to other factor(s). To investigate this further, the mutated *codY* in strain Δ*codY* was replaced with a wild-type *codY* allele to generate strain YS18 (Fig. 1A). YS18 also displayed natural competence (Fig. 1B), confirming that a mutation, rather than the loss of CodY function, is responsible for the competent phenotype in both strains Δ*codY* and YS18. As σ^X^ is the master regulator for expressing competence in streptococci (6), we first assessed *comX* expression in strains 57.I, Δ*codY*, and YS18 by RT-qPCR. Both strains Δ*codY* and YS18 exhibited a more than 20-fold increase in *comX* expression compared to strain 57.I (Fig. 1C). Western blot analysis using an anti-ComX polyclonal antibody confirmed that ComX protein was highly produced in Δ*codY* and YS18 but was undetectable in the wild-type 57.I (Fig. 1D).

**Fig 2:**
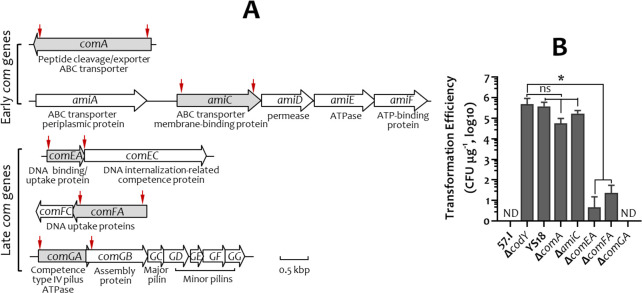
The impact of early and late *com* genes on the competence of *S. salivarius* Δ*codY*. (**A**) Schematic presentation of selected *com* genes/operons. The proposed function of each gene is listed below the gene. Genes insertionally inactivated by Ω*kan* are shaded. The deleted region of each gene is indicated by two vertical red arrows. (**B**) Transformation efficiency of *S. salivarius* strains. The non-competent 57.I and competent Δ*codY* were used as negative and positive controls, respectively. Transformation efficiency is expressed as CFU μg^−1^ pDL278 in log_10_. The numbers are the mean and standard deviation of five biological samples. ND, not detected. Significant differences between the *com*-deletion mutant strains and the control Δ*codY* were analyzed using an unpaired Student’s *t*-test. *, *P* < 0.05; ns, not significant.

The above results suggested that Δ*codY* and YS18 either possess an activated early Com signaling pathway or that *comX* is constitutively expressed in these strains. To distinguish between these two possibilities, the early and late *com* genes in *S. salivarius* were identified based on their homology to counterparts in *S. pneumoniae* (a ComAB-CDE system user) and *S. thermophilus* (a ComRS-Aim system user) (Table S2). Among these *com* genes, two early *com* genes, *comA* and *amiC*, and three late *com* genes, *comEA*, *comFA*, and *comGA*, were insertionally inactivated by Ω*kan* in *S. salivarius* Δ*codY* (Fig. 2A). Competence analysis revealed that inactivation of the early *com* genes, *comA* and *amiC*, did not abolish natural competence. On the other hand, deficiency in the late *com* genes (*comEA*, *comFA*, and *comGA*) severely reduced competence (Fig. 2B). Similarly, inactivation of *comA* did not affect the competence of YS18, whereas deletion of *comGA* abolished the competence of YS18 (Fig. S1). Thus, *comX* expression in strains Δ*codY* and YS18 was independent of the induction by the early Com system.

### Functional analysis of p*_comGA_*

**Fig 3:**
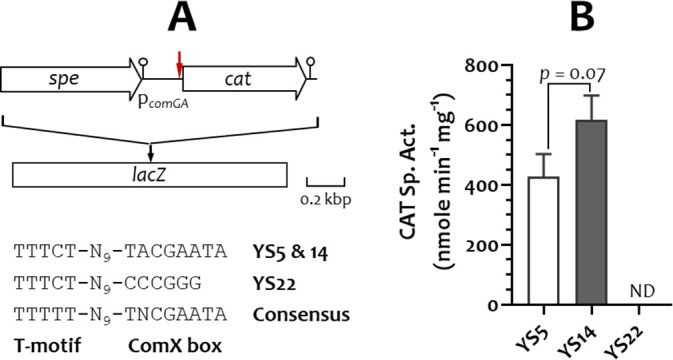
Expression analysis of the *comGA* operon promoter (p*_comGA_*) in *S. salivarius* strains. (**A**) Schematic of the *spe-*tagged p*_comGA_-cat* integration in *S. salivarius lacZ*. The putative transcription terminator is indicated by a lollipop, and the ComX box location is indicated by a vertical red arrow. ComX box sequences in YS5, YS14, and YS22, along with the consensus sequence, are shown. (**B**) CAT-specific activity of the p*_comGA_-cat* fusion strains. Values are the means and standard deviations from three independent experiments. ND, not detected. Significant differences between samples were analyzed using an unpaired Student’s *t*-test.

A p*_comGA_-cat* fusion was integrated into the *lacZ* locus of *S. salivarius* Δ*codY* and YS18 to assess ComX-dependent activation of late *com* gene expression (Fig. 3A). Approximately 400 U and 600 U of CAT activity were detected in the Δ*codY* (strain YS5) and YS18 (strain YS14) hosts, respectively (Fig. 3B), which is consistent with ComX-dependent promoter activation. To verify that the observed CAT activity was specifically mediated by ComX, the predicted ComX-binding site (ComX box, TACGAATA) in p*_comGA_-cat* was replaced with CCCGGG in YS5, generating strain YS22. No CAT activity was detected in YS22, confirming that p*_comGA_* activation depends on ComX. This finding further supports that ComX regulates the expression of all late *com* genes containing the conserved ComX-box motif.

### 5′ RACE analysis of YS18 *comX*

**Fig 4:**
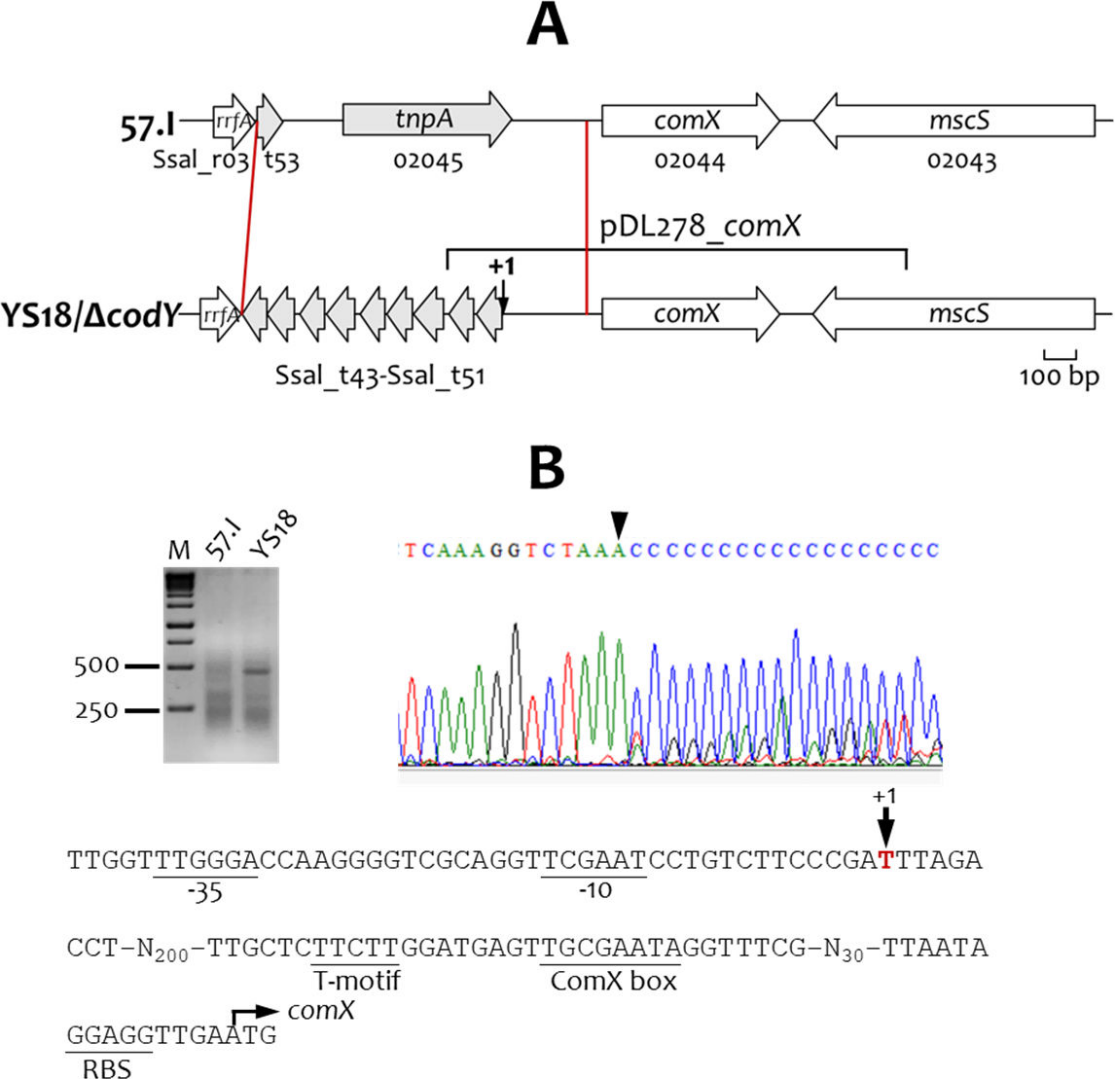
The *comX* locus in *S. salivarius* 57.I, Δ*codY*, and YS18. (**A**) Schematic diagram of *comX* and its flanking regions. Gene names and Ssal tag numbers are shown. The two red lines indicate the region that differs between strains, with genes colored gray; whereas, genes conserved in this region are colored white. The region used for constructing pDL278_*comX* is indicated by a horizontal half bracket above the map. (**B**) Identification of the transcription initiation site of *comX* in YS18 by 5′ RACE. The PCR amplicon is shown in the upper left. M, 100 bp marker. Sequencing analysis of the PCR products is shown on the upper right. The transcription initiation site (+1) is indicated by an inverted triangle. The 5′ flanking sequence of YS18 *comX* is shown below. The putative −35 and −10 elements, the +1 site, the ribosomal binding site (RBS), the ComX box, and the T-motif are indicated.

To identify element(s) allowing the constitutive expression of *comX*, the 5′ flanking regions of *comX* in Δ*codY* and YS18 were sequenced and compared with those of 57.I (GenBank accession number CP002888.1). The result indicated that a 987 bp region of 57.I, including Ssal_t53 and Ssal_02045 (*tnpA*), was replaced with 9 tRNA coding sequences in Δ*codY* and YS18 (Fig. 4A). Next, the transcription initiation site of *comX* in YS18 was analyzed by 5′ RACE. One signal, mapped to a T, was detected 288 bases 5′ to the ATG (Fig. 4B). A σ^70^-type promoter (TTGGGA-N_16_-TCGAAT) was observed 14 bases 5′ to the +1 site. A putative ComX box (TNCGAATA) and the conserved T-motif (21, 22) were found 53 bases 5′ to the translation start site, suggesting that ComX may further enhance its own expression through a positive feedback loop. Interestingly, the proposed ComR binding consensus (19, 20) was not observed in the promoter region. Although the *comX-*specific message was detected in 57.I by qPCR (Fig. 1C), no quantifiable PCR product could be obtained in 5′ RACE with RNA isolated from strain 57.I (Fig. 4B), indicating a low abundance of *comX*-specific message in 57.I.

### *com* genes in *S. parasanguinis* FW213

BlastP analysis indicated homologs of the early and late *com* genes in *S. salivarius* 57.I and *Streptococcus sanguinis* SK36 were also present in the genome of *S. parasanguinis* FW213 (Table 2). Notably, the sequences of all *com* genes are identical in *S. salivarius* 57.I, Δ*codY*, and YS18. Thus, although FW213 is non-competent, it harbors the complete set of *com* genes found in naturally competent *S. salivarius* and *S. sanguinis*. Among the Com proteins, limited homology was observed in the ComAB-ComCDE system between strains (24%–55% identity and 49%–77% similarity), especially ComA. Furthermore, although homologs of the Ami transporter components were identified in *S. sanguinis* SK36 and *S. parasanguinis* FW213, neither strain harbors genes encoding the ComRS system, indicating that ComCDE is the key QS system for initiating competence development in S. *sanguinis*. On the other hand, higher levels of homology (35%–81% identity and 56%–97% similarity) were observed in the late *com* genes among strains, reflecting conservation of the DNA uptake systems for natural transformation. Similarly, the proposed DNA processing enzymes were also conserved (46%–79% identity and 63%–87% similarity) between strains. Thus, it was hypothesized that FW213 fails to activate competence expression rather than lacking the essential late *com* genes for DNA incorporation.

**TABLE 2:** Putative *com* genes in *S. salivarius* 57.I, *S. sanguinis* SK36, and *S. parasanguinis* FW213

Gene name^*a*^	Function^*b*^	Homolog^*c*^		
		*S. parasanguinis* FW213	*S. salivarius* 57.I	*S. sanguinis* SK36
Com AB-CDE QS system				
*comA*	Process and export CSP	Spaf_1084	Ssal_01906, 24% (49%)	SSA_1100, 27% (50%)
*comB*	Process and export CSP	–	–	–
*comC*	CSP	–	–	–
*comD*	Histidine kinase	Spaf_0318	Ssal_01705, 41% (63%)	SSA_2379, 28% (58%)
*comE*	Response regulator	Spaf_0317	Ssal_01706, 55% (77%)	SSA_2378, 38% (58%)
ComRS-Ami QS system				
*comR*	Regulator of the XIP QS system	–	Ssal_01907	–
*comS*	XIP procusor	–	–	–
*amiA*	XIP uptake system, substrate-binding protein	Spaf_1301	Ssal_00622, 58% (75%)	SSA_1948, 57% (76%)
*amiC*	XIP uptake system, membrane-binding protein	Spaf_0530	Ssal_00624, 72% (85%)	SSA_1947, 76% (87%)
*amiD*	XIP uptake system, membrane-binding protein	Spaf_0531	Ssal_00625, 79% (90%)	SSA_1946, 85% (93%)
*amiE*	XIP uptake system, ATP-binding protein	Spaf_0532	Ssal_00626, 83% (93%)	SSA_1945, 87% (93%)
*amiF*	XIP uptake system/ATP-binding protein	Spaf_0533	Ssal_00627, 84% (93%)	SSA_1944, 86% (94%)
Sigma factor				
*comX*	Competence-specific σ^X^	Spaf_0014	Ssal_02044, 35% (63%)	SSA_0016, 53% (71%)
DNA uptake system				
*comEA*	DNA binding and uptake	Spaf_0704	Ssal_00493, 44% (64%)	SSA_0715, 55% (71%)
*comEC*	DNA internalization-related competence protein	Spaf_0705	Ssal_00495, 48% (67%)	SSA_0716, 56% (73%)
*comEB*	DNA binding and uptake	Spaf_0842	Ssal_00281, 55% (69%)	SSA_1497, 81% (97%)
*comFC*	DNA translocase	Spaf_0240	Ssal_01822, 47% (62%)	SSA_1835, 49% (69%)
*comFA*	DNA translocase	Spaf_0239	Ssal_01823, 49% (69%)	SSA_1836, 57% (77%)
*comGA*	ABC transporter subunit	Spaf_0284	Ssal_00142, 66% (78%)	SSA_0184, 75% (88%)
*comGB*	ABC transporter subunit	Spaf_0286	Ssal_00143, 52% (73%)	SSA_0185, 71% (83%)
*comGC*	Major pilin	Spaf_0287	Ssal_00144, 71% (87%)	SSA_0186, 72% (91%)
*comGD*	Minor pilin	Spaf_0288	Ssal_00145, 41% (68%)	SSA_0187, 50% (71%)
*comGE*	Minor pilin	Spaf_0289	Ssal_00146, 37% (61%)	SSA_0188, 53% (70%)
*comGF*	Minor pilin	Spaf_0290	Ssal_00147, 43% (64%)	SSA_0189, 62% (77%)
*comGG*	Minor pilin	Spaf_0291	Ssal_00148, 62% (73%)	SSA_0190, 35% (56%)
DNA processing enzymes				
*coiA*	Competence protein CoiA	Spaf_0724	Ssal_01737, 46% (63%)	SSA_0749, 51% (67%)
*smf*	DNA processing Smf protein	Spaf_1071	Ssal_01014, 63% (79%)	SSA_1185, 65% (81%)
*cinA*	Competence-damage inducible protein	Spaf_2032	Ssal_02112, 73% (83%)	Ssal_02112, 73% (83%)
*ssb*	ssDNA-binding protein	Spaf_0304	Ssal_00163, 76% (87%)	SSA_0214, 79% (87%)

^*a*^ Genes encoding proteins involved in competence development in *S. pneumoniae* or *S. thermophilus* are listed.

^*b*^ Proposed functions are based on the annotation of *S. pneumoniae *or S*. thermophilus* Com proteins.

^*c*^ Homologs in *S. parasanguinis* FW213 (Spaf), *S. salivarius* 57.I (Ssal), and *S. sanguinis *SK36 (SSA) are shown. Percent identity and similarity (in parentheses) are shown for the Spaf locus relative to Ssal and SSA loci. –, not identified.

### Effect of *S. salivarius* ComX on competence induction in *S. parasanguinis* FW213

**Fig 5:**
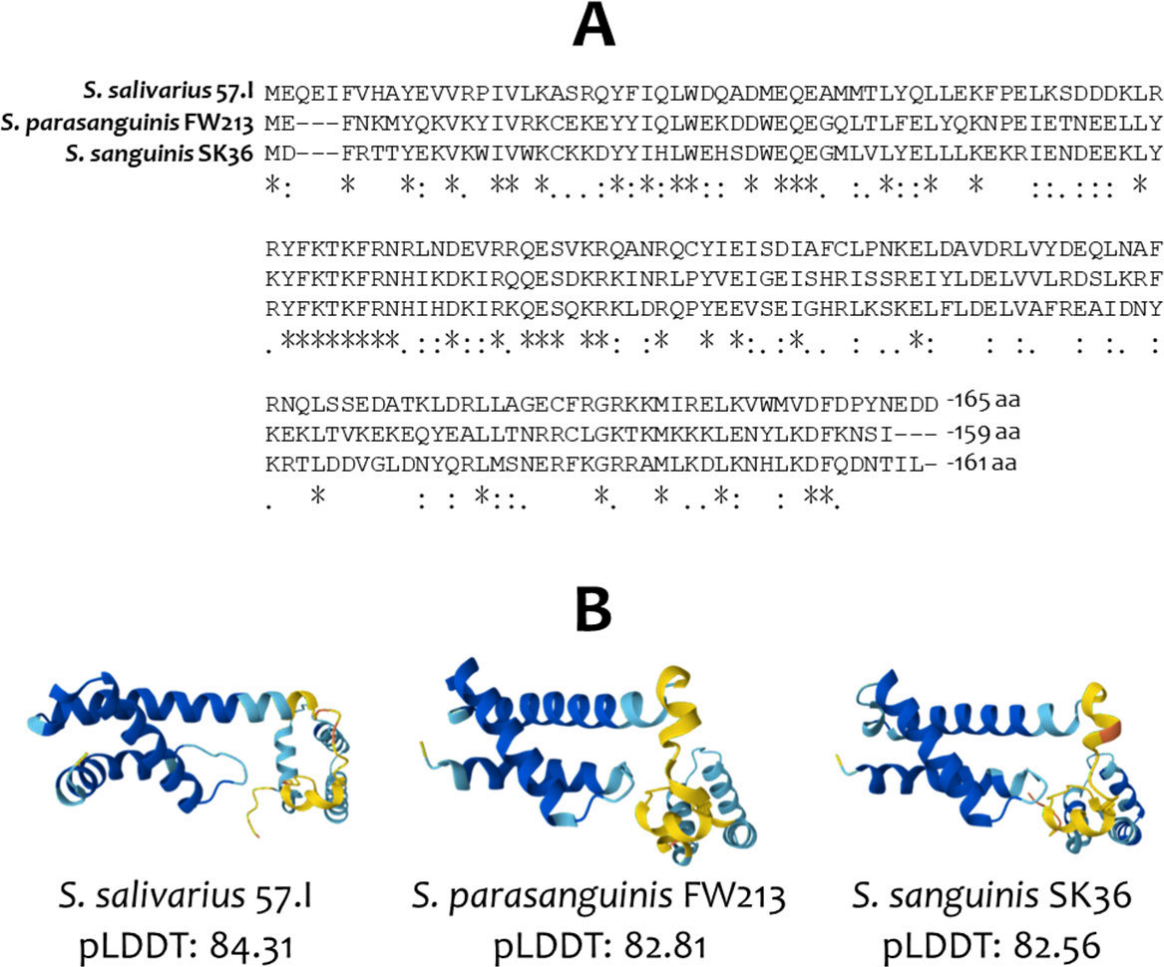
Sequence and structural conservation of ComX between *S. salivarius* 57.I, *S. parasanguinis* FW213, and *S. sanguinis* SK36. (**A**) Alignment of the ComX proteins. Identical residues are indicated by stars, conserved substitutions by colons, and less conserved substitutions by dots. (**B**) AlphaFold-predicted 3D structure. The level of model confidence scores (pLDDT) is indicated by color: cobalt blue, pLDDT > 90; sky blue, pLDDT > 70; yellow, pLDDT > 50; orange, pLDDT < 50. The average pLDDT for each ComX protein is shown.

**Fig 6:**
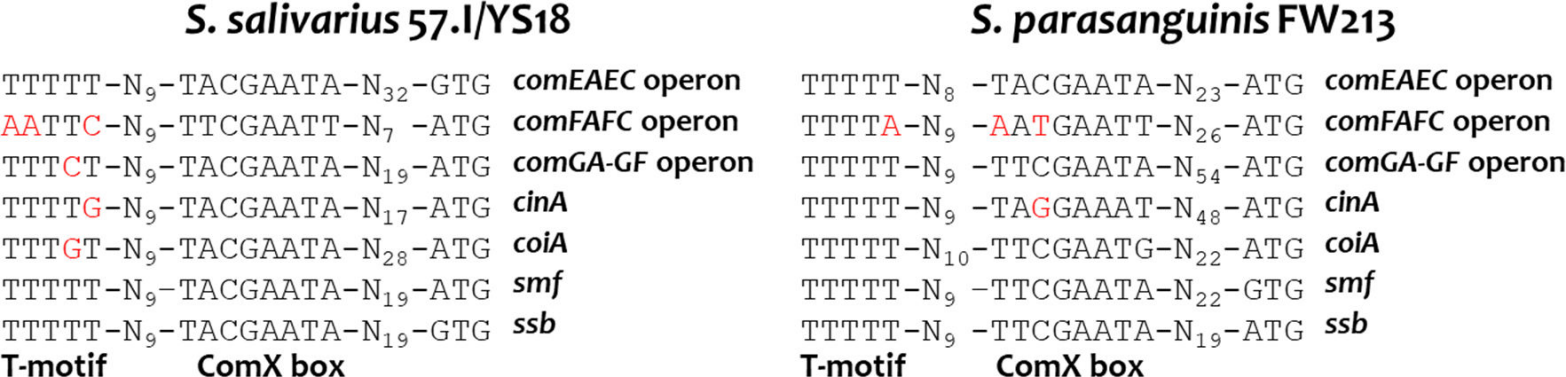
The promoter regions of the ComX-regulated genes in *S. salivarius* 57.I/YS18 and *S. parasanguinis* FW213. The conserved ComX boxes and TTTTT motifs are aligned. The distance between each element is indicated. Nucleotides colored red deviate from the consensus.

The above sequence analysis raises the possibility that a constitutively expressed ComX could initiate competence development in *S. parasanguinis* FW213. Although the putative FW213 ComX shares only 35% and 53% amino acid identity with its orthologs in *S. salivarius* 57.I and *S. sanguinis* SK36, respectively, multiple sequence alignment revealed conservation across the entire protein sequence (Fig. 5A). AlphaFold prediction further indicated that the tertiary structures of these ComX proteins are highly similar (Fig. 5B), supporting the possibility of functional compatibility. Additionally, when examining the 5′ flanking sequence of the putative late *com* genes of *S. salivarius* 57.I/YS18 and *S. parasanguinis* FW213, a ComX box and a conserved T-motif in the appropriate location (42) were detected in the 5′ flanking regions of the *comGA-GF* operon, *comEAEC* operon, *comFAFC* operon, *cinA*, *coiA*, and *ssb* in both species (Fig. 6), supporting the notion that ComX is involved in the transcription of these genes.

**Fig 7:**
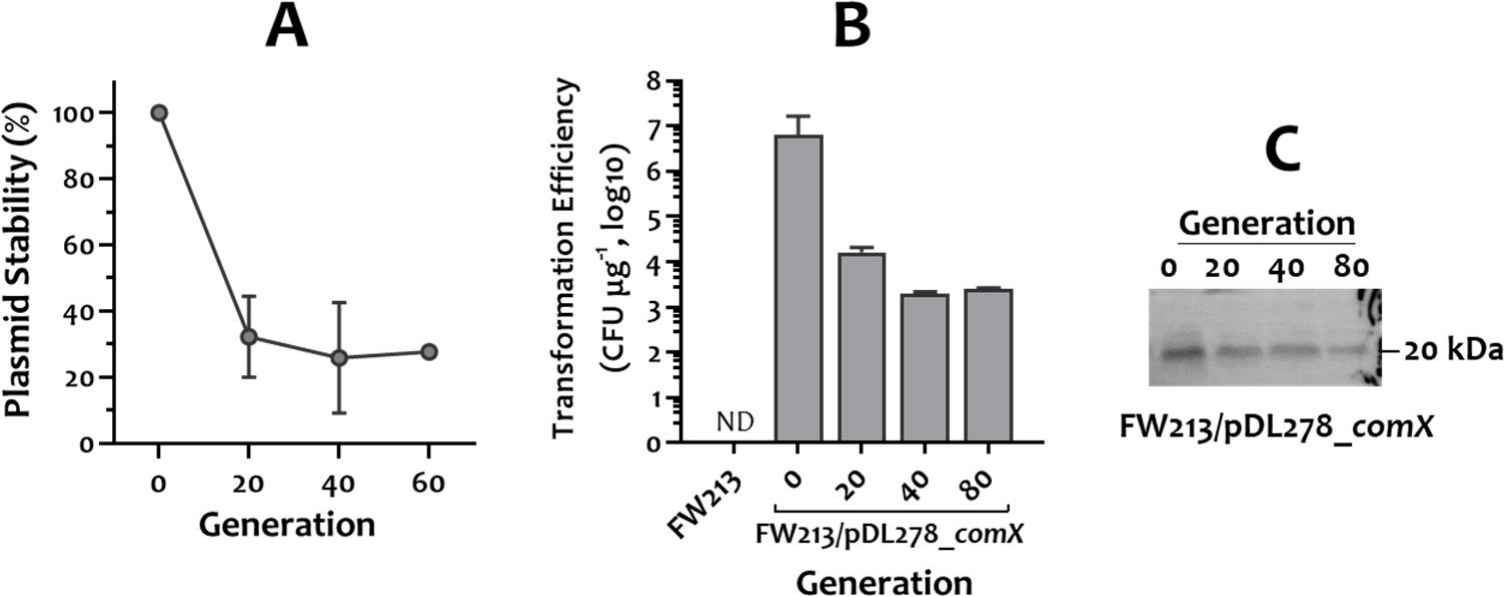
The effect of ComX from *S. salivarius* Δ*codY*/YS18 in *S. parasanguinis* FW213 competence. (**A**) Stability of pDL278_*comX* in *S. parasanguinis* under nonselective conditions. The numbers are the mean and standard deviation of three independent samples. (**B**) Transformation efficiency of wild-type *S. parasanguinis* FW213 and FW213 harboring pDL278_*comX*. The plasmid-containing strain was passaged daily in plain TH, and the transformation efficiency at each passage is expressed as CFU μg^−1^ of pDL276. ND, not detected. (**C**) Western blot showing ComX protein levels in *S. parasanguinis* over the course of passages.

To test whether the constitutively expressed ComX of *S. salivarius* Δ*codY*/YS18 could induce the competence of *S. parasanguinis* FW213, the *comX* gene of *S. salivarius* Δ*codY*/YS18, with its promoter region (Fig. 4A), was cloned into pDL278 and established in *S. parasanguinis* FW213. Plasmid pDL278 (33) contains a pVA380-1 replicon and has been widely used in many streptococcal strains. After introducing pDL278_*comX* into FW213 by electroporation, induced competence was observed with a transformation efficiency at 6.3 × 10^6^ CFU µg^−1^ (Fig. 7B).

Plasmid pDL278_*comX* was unstable without selection (Fig. 7A). In the absence of selection, transformation efficiency declined progressively (Fig. 7B), paralleling the reduced ComX production (Fig. 7C), indicating that the plasmid-borne *comX* can transiently activate competence in *S. parasanguinis* FW213. Thus, it is feasible to generate mutant strains in FW213 via ligation mutagenesis, a transformation-based mutagenesis approach (29), during the temporary competence state.

Although the function of the constitutively expressed *comX* of Δ*codY*/YS18 in competence activation in *S. parasanguinis* FW213 was evident, it remained possible that additional mutations in *S. salivarius* YS18 contribute to the competence phenotype. To verify whether the *comX* allele from Δ*codY*/YS18 alone could induce competence in *S. salivarius*, we also examined the competence of *S. salivarius* 57.I harboring pDL278_*comX*. The recombinant strain exhibited a transformation efficiency of 7.19 × 10^3^ CFU µg^−1^, confirming that the mutation in the 5′ flanking region of *comX* was sufficient to confer the competence phenotype (Fig. S2). However, the transformation efficiency of YS18 was approximately 17 times higher than that of 57.I harboring pDL278_*comX*. While the basis for this difference is unclear, it may suggest that the genomic context of YS18 provides more optimal expression levels than 57.I.

### Inactivation of loci encoding membrane proteins in *S. parasanguinis* FW213

**Fig 8:**
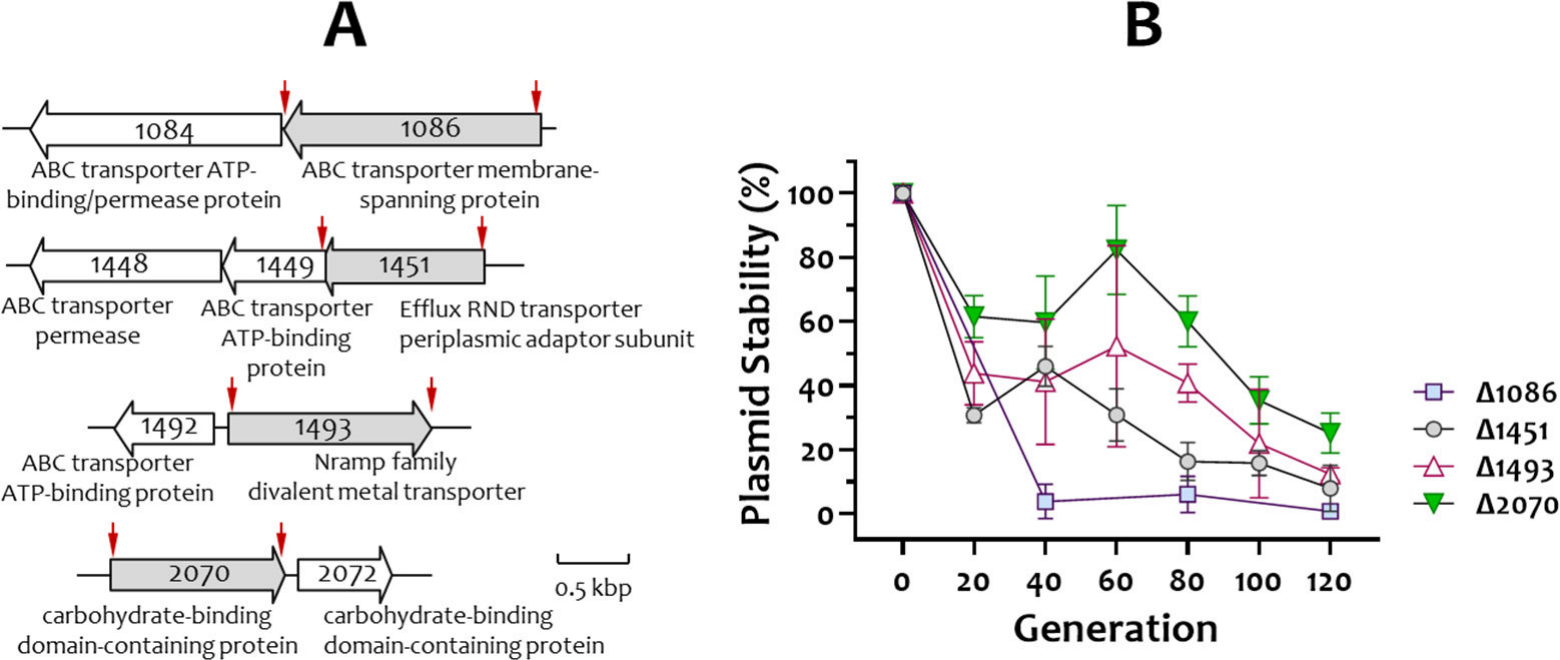
Mutant construction and stability of the *comX*-containing plasmids in *S. parasanguinis* FW213. (**A**) Schematic presentation of selected membrane protein-encoding loci in *S. parasanguinis* FW213. Spaf tag numbers are indicated. The proposed function is listed below the gene. Genes that were insertionally inactivated by *erm* are shaded, and the deleted region is indicated by two vertical red arrows. (**B**) Stability of pDL278_*comX* in the recombinant *S. parasanguinis* mutant strains under non-selective conditions.

Genes encoding membrane proteins are often unstable on high-copy-number plasmids in *E. coli*, making it difficult to obtain sufficient, high-quality plasmid DNA for downstream mutagenesis. To evaluate the effectiveness of the induced competence system for generating targeted mutant strains of *S. parasanguinis* FW213, we selected four loci encoding membrane proteins for inactivation by ligation mutagenesis. Allelic exchange in the selected loci was carried out by replacing the coding sequence with the *erm* resistance cassette (Fig. 8A). Mutants were successfully obtained in FW213 harboring pDL278*_comX*. After confirmation of allelic exchange, recombinant isolates were passaged in antibiotic-free TH medium to assess plasmid stability. While the frequency of loss of *comX*-containing plasmids varied among isolates (Fig. 8B), Sp-sensitive isolates were easily obtained. PCR analysis confirmed the loss of *comX*-containing plasmids, verifying the efficiency and utility of the system for generating stable mutants in previously non-competent *S. parasanguinis* FW213.

## DISCUSSION

Many streptococcal species/strains harbor a complete set of *com* genes but fail to express competence (43, 44), including *S. salivarius* 57.I and *S. parasanguinis* FW213, both of which have been studied by multiple research groups for many years. However, efforts to identify conditions that induce competence have consistently been unsuccessful. In our laboratory, we have systematically tested various conditions, including cultures at different O.D._600_ values (O.D._600_ = 0.05, 0.2, and 0.4), in complex medium BHI or chemically defined medium FMC, with the addition of varying amounts of horse serum. However, none of these treatments resulted in detectable competence in both FW213 and 57.I under our experimental conditions. Thus, the mechanism by which to activate the early *com* system in *S. salivarius* 57.I and *S. parasanguinis* FW213 remains unclear.

As defects in CSP/XIP signaling or insufficient production of CSP/XIP can limit competence development, approaches such as supplementation of exogenous CSP/XIP have been successfully used to induce or enhance competence in various streptococcal species (45–48). However, CSP/XIP is often highly species- or strain-specific. Specifically, XIP exhibits substantial size and primary sequence variation among streptococcal species (49), and polymorphism is observed in CSP among *Streptococcus mitis* strains and *S. pneumoniae* (9). As *S. salivarius* mainly utilizes the ComRS system for competence expression, an attempt was made previously to use a synthetic peptide corresponding to the amino acids 18–24 of *S. thermophilus* LMD-9 ComS to induce competence in *S. salivarius* 57.I. This peptide was able to induce the competence of *S. thermophilus* CNRZ1066, *S. salivarius* ATCC25975 and 8770, all of which are otherwise non-competent (10). However, the same peptide failed to induce competence in *S. salivarius* 57.I (data not shown). Since we did not detect nonsense mutations in genes encoding the Ami peptide uptake system, the proposed ComRS, nor the late Com proteins, it is suggested that *S. salivarius* 57.I either utilizes a different XIP or the induced *comX* expression is insufficient to initiate the expression of the late *com* genes.

A competence-activation threshold for ComX was evident in *S. salivarius* 57.I (Fig. 1). To increase *comX* expression in non-competent *S. salivarius* 57.I, we initially employed a strong streptococcal promoter, p*ureI*Δ21 (50), to drive *comX* expression. Although the strain harboring the p*ureI*Δ21-*comX* fusion on the chromosome exhibits a fourfold increase in *comX* expression compared to the parental 57.I, the cells remain non-competent, indicating that competence initiation requires *comX* expression to surpass a critical level. By employing a plasmid-based *comX* expression system based on the *comX* locus from *S. salivarius* Δ*codY*/YS18, a substantially high amount of ComX was obtained, enabling more robust activation of the competence regulon.

Previous studies have similarly demonstrated that an increased intracellular amount of the competence sigma factor can trigger competence development. For instance, in *Staphylococcus aureus*, duplication of *sigH* led to elevated sigma factor production and activation of the competence regulon (51), whereas in *Lactococcus lactis*, controlled upregulation of *comX* expression was sufficient to enable natural competence (52). Our findings are consistent with these observations, suggesting that enhanced *comX* expression positively influences competence development. Additionally, our preliminary transcriptomic analysis revealed comparable expression levels in genes encoding the competence QS systems between non-competent 57.I and competent YS18. However, YS18 exhibited 2^6^ to 2^10^-fold upregulation in the late *com* genes compared to 57.I, highlighting the critical amount of ComX necessary for competence activation. These findings suggest that the failure to produce enough ComX, rather than a lack of competence QS systems, may account for the non-competent phenotype in many *com* gene-containing, yet non-competent, streptococci.

Although sequence homology is useful in identifying homologs, variations of CSP (1) and the structural conservation of ABC transporters complicate the reliable identification of the Com-specific signaling system without functional analysis. For instance, the proposed ComA of *S. salivarius* (Ssal_01906) was based on its 72% similarity to *S. pneumoniae* ComA. However, only loci with less than 30% identity are present in *S. sanguinis* and *S. parasanguinis* (Table 2), and neither of these species harbors a ComB homolog. Thus, whether the proposed ComA truly participates in competence development is unclear. In *S. sanguinis*, ComC and its export system ComAB remained undefined for years, as the proposed CSP lacked the Gly-Gly motif required for ComA recognition and cleavage. A recent study in *S. sanguinis* VMC66 (53) indicated that ComC is exported and processed by two non-ComAB ABC transporters (94247 and 94977), which also allows for a direct link between competence and other cellular activity, such as bacteriocin production. Interestingly, only the 94977 homolog is found in the *S. sanguinis* SK36 genome (98% identity), and homologs of both transporters are absent in *S. salivarius* 57.I/YS18 and *S. parasanguinis* FW213, reflecting the complexity of the early Com system. Likewise, low-identity homologs of *S. salivarius* Ami transporter proteins could be identified in the genomes of *S. parasanguinis* and *S. sanguinis* (Table 2), even though XIP is unknown in Mitis streptococci. In contrast, the conservation of DNA and processing uptake systems across streptococci indicates a common DNA acquisition machinery. Consequently, with the structural conservation of ComX, we reasoned that competence could be activated through overexpression of *comX*, effectively bypassing the early Com signaling systems.

In summary, as transient competence can provide specific advantages for bacteria, it remains unknown why *com* gene-containing streptococcal strains, such as *S. salivarius* 57.I and *S. parasanguinis* FW213, would evolve into a non-competent phenotype. Nevertheless, ComX abundance is the central determinant of competence development, and rather than malfunction of the QS systems, limited ComX production likely underlies the non-competent phenotype observed in many streptococcal species and strains.

## Data Availability

The data supporting the findings of this study are available within the article and its supplemental material. Materials and additional data supporting the conclusions of this study will be made available by the corresponding author to qualified researchers upon reasonable request for noncommercial purposes.

## SUPPLEMENTAL MATERIAL

Supplemental material aem.00082-26-s0001.pdf Tables S1 and S2; Fig. S1 and S2.

ASM does not own the copyrights to Supplemental Material that may be linked to, or accessed through, an article. The authors have granted ASM a non-exclusive, world-wide license to publish the Supplemental Material files. Please contact the corresponding author directly for reuse.
