# Supplementary material for: A ComX-based strategy for artificially inducing competence in naturally non-transformable Streptococcus parasanguinis
Source: Appl Environ Microbiol. 2026 Mar 18;92(4):e00082-26. doi: 10.1128/aem.00082-26 (PMC13101493; doi:10.1128/aem.00082-26)
Supplement: Supplemental material — Tables S1 and S2; Fig. S1 and S2. [file aem.00082-26-s0001.pdf]

Table S1. Primer used in this study.

Primer	Sequence[a]	Purpose
ala_488_S	GGTGAGCTCGTTTCAGAGATGATTTCCATGT	Construction of *S. salivarius* YS18
ala_1390_BamHI_AS	AGGGATCCAAGAAATTAACGCTTGTAATATG	
codY_815_BamHI_S	TTGGATCCCTGGACAAAAAGGCTTGTCC	
codY_3606_SphI_AS	ATAGCATGCCTTGTGACATTCTTTGAAGAGG	
comX_1025	CGAAGACAACTGATTACGGAAAGC	*comX* qPCR
comX_1236	GTCTGATGATGATAAGCTACGGCG	
comX_BamHI_S	GGAGGATCCATGGAACAAGAAATTTTTGTT	Construction of pQE30/*comX*
comX_SphI_AS	TATGCATGCTTAATCATCTTCATTGTAAGG	
0141_3465_SmaI_S	AAGCCCGGGTGTGAGGAGTGTGATGAATACC	Generation of p_{comGA} fragment
GA_3679_SalI_AS	CATGTCGACCTCCTCATAGACCTATTCGTAAA	
lacZ_S	GGTTTTGGTTCTCCACAATATGTG	ComX box replacement in p_{comGA}-*cat*
Δbox_GA3662_AS	GGGAAAAGTTAAAGAAAATGAAAAAAGCTC	
Δbox_GA3671_SmaI_S	GGGGGTCTATGAGGAGGTCGACATG	
lacZ_AS	CAGCAACATATTGACCGCGAAC	
lacS_1241	GGGATATCAAAGTGATGAAAC	Confirmation of integration at *lacZ*
lacAS_1465	GGCACGATCCAAACAAGC	
comX_4470_AS	CTATCAACAGCATCTAACTCTTT	5' RACE
comX_5143_AS	GCTTGTCGTTTTACAGACTCC	
ComX_5022_AS	GCTGATAAAGCGTCATCATAGC	
ComX_3701_BamHI_S	ATAGGATCCCACCCTTAGCTCAACTGGATAG	Generation of *comX* fragment
comX_5029_XhoI_AS	ATACTCGAGATCCCAGTCTATCCATCAACCG	
comA_2345_S	CCAAGCCAGCATTCCAGCCC	Construction of *S. salivarius* Δ*comA*
comA_SphI_3294_AS	TGGCATGCGGTCAGATTGAGTCGGACAGTCC	
comA_XhoI_5201_S	GCCTCGAGGCCTTCACGAGAAGTCTGAGCC	
comA_6377_AS	CTGAGCGTGAACGTCAGCAGATG	
amiC_6415_S	GCTGCCGCAGAGGATTGGG	Construction of *S. salivarius* Δ*amiC*
amiC_SphI_7532_AS	TAGCATGCGGTGTAGGTCAAGGCCGTCACG	
amiC_XhoI_8868_S	AGCTCGAGCCATCTTCTCATTGCTTGTGGG	
amiC_9864_AS	CGTGGGTCGCTGGCATCCGC	
comEA_1505_S	GCTGCAATGCTGTGGTTACTACC	Construction of *S. salivarius* Δ*comEA*
comEA_SphI_2631_AS	GCGCATGCCTGCGTTTCTTTGGCACGGTG	
comEA_XhoI_3311_S	GGCTCGAGGCGTGCTGAGGATATCATTGCC	
comEA_4223_AS	GGAAGGCCCAAGCGTAGGAGT	
comFA_5_S	AGAACACTTCTCCTGTTGGAACT	Construction of *S. salivarius* Δ*comFA*
comFA_SphI_1008_AS	ACGCATGCCCAGAGAGAAGAGTACTCCTGC	
comFA_XhoI_2226_S	CGCTCGAGGCCAATCTTCCTCTATCTGACTG	
comFA_3324_AS	CAGCATCTTCAATCATACGAAGAG	
comGA_2642_S	CGGTGACAAACACGTCGAAGTAA	Construction of *S. salivarius* Δ*comGA*
comGA_3708_AS_SphI	TAGCATGCTCTGTTACCATAAGGACCTCCTC	
comGA_4588_S_XhoI	ATCTCGAGGAAGGGGATATCCAAGGGGA	
comGA_5693_AS	GATAATGAGAAGCACGACCAGC	
spaf1086_L_S4782	GTGCTCCAAGTTCAAGTGTTGATGC	

spaf1086_L_AS3822SphI	AAGCATGCTCTGCCCCAGTCCCATCTGTAGT	Construction of *S. parasanguinis* Δ1086
spaf1086_R_S2028BamHI	AAGGATCCCGATAGTCCATCATTGACAGAAGAATAGAA	
spaf1086_R_AS1252	TTTGAAGGTGATGGGTAATATCATGAA	
spaf1451_L_AS_2033SphI	ATGCATGCCGTTTTTCTCCATCAAAGAAAGCGTTTT	Construction of *S. parasanguinis* Δ1451
spaf1451_L_S_1129	TTCACACGACCGTCTTCTAATTTTTC	
spaf1451_R_S_3189BamHI	ATGGATCCTGAACTAAAAGGAATCAATAAAACCTATA	
spaf1451_R_AS_4090	ATATTGATTCCTGTCACATAGCCA	
spaf1493_L_S1273	GAATCACACGGTCTGCAATCGGAGCTA	Construction of *S. parasanguinis* Δ1493
spaf1493_L_AS2015SphI	TTGCATGCTTTATAATAGTTCATGATAATTTGGGGTTA	
spaf1493_R_S3392BamHI	GGGGATCCTGATTGTGACAACTTTTATCAAATCATGACCA	
spaf1493_R_AS4159	TCAGTCCGTCTCGCTCCGTTAGGT	
spaf2070_L_S_2361	GATGACCATAACGTTTGCGCAATC	Construction of *S. parasanguinis* Δ2070
spaf2070_L_AS_2977XhoI	AAGCATGCGAGCTTTTCCTCTCGTTCAATTGA	
spaf2070_R_S_4228BamHI	AAGGATCCTAATAACAAATAAAGGCAGGATTTCTTCC	
spaf2070_R_AS_5125	GGATTGTCGAGCAAGACGCAGTGGT	
erm_S	GTGATCGATTCACAAATCACTTATCACAAATCA	Generation of the *erm* fragment
erm_AS	CTGGGATCCACAAATTCCCCGTAGGCGCTAG	
kan_S	GTGCATGCTCGATAAGCTTGGATCC	Generation of the Ω*kan* fragment
kan_AS	CGCTCGAGGCTCTCCGGATCC	
spe_S	GTCGATTTTCGTTCGTGAATACAT	Generation of the *spe* fragment
spe_AS	TTAGACTCGAGGAATTCGG	

a, restriction sequences are underlined.

Table S2. *com* genes in *S. salivarius* 57.I/YS18

Gene	Gene-ID[a]	Homologs[b]	Proposed function	Identity[c]	Similarity[d]
Early *com* genes					
comA	Ssal_01906	*S. pneumoniae comA*	CSP processing and transport	369/691 (53%)	504/691 (72%)
comB	-		CSP processing and transport		
comC	-		CSP		
comD	Ssal_01705	*S. pneumoniae* R6 *comD*	histidine kinase of ComDE	90/275 (33%)	159/275 (57%)
comE	Ssal_01706	*S. pneumoniae* R6 *comE*	response regulator of ComDE	86/234 (37%)	138/234 (58%)
comR	Ssal_01907	*S. thermophilus* LMD-9 *comR*	transcriptional regulator of ComRS	260/280 (93%)	275/280 (98%)
comS	NA	*S. thermophilus* LMD-9 *comS*	XIP	18/24 (75%)	20/24 (83%)
amiA	Ssal_00622	*S. thermophilus* LMG 1831 *amiA3*	oligopeptide ABC transporter, substrate-binding protein	486/657 (74%)	570/657 (86%)
amiC	Ssal_00624	*S. thermophilus* LMG 18311 *amiC*	oligopeptide ABC transporter, membrane-binding protein	484/497 (97%)	490/497 (98%)
amiD	Ssal_00625	*S. thermophilus* LMG 18311 *amiD*	oligopeptide ABC transporter, permease	300/308 (97%)	305/308 (99%)
amiE	Ssal_00626	*S. thermophilus* LMG 18311 *amiE*	oligopeptide transport, ATP-binding protein	332/346 (96%)	342/346 (98%)
amiF	Ssal_00627	*S. thermophilus* LMG 18311 *amiF1*	oligopeptide transport, ATP-binding protein	304/309 (98%)	307/309 (99%)
Sigma factor					
comX	Ssal_02044	*S. thermophilus* LMG 18311 *comX*	sigma X	145/165 (88%)	154/165 (93%)
Late *com* genes					
comEA	Ssal_00493	*S. thermophilus* LMG 18311 *comEA*	DNA binding and uptake	209/231 (90%)	219/231 (94%)
comEC	Ssal_00495	*S. thermophilus* LMG LMD-9 *comEC*	DNA internalization-related competence protein	654/746 (88%)	702/746 (94%)
comEB	Ssal_00281	*S. thermophilus* CNRZ1066 *comEB*	DNA binding and uptake	146/151 (97%)	148/151 (98%)
comFA	Ssal_01823	*S. thermophilus* LMG 18311 *comFA*	DNA uptake	377/439 (86%)	405/439 (92%)
comFC	Ssal_01822	*S. thermophilus* LMG 18311 *comFC*	DNA uptake	123/156 (79%)	143/156 (91%)
comGA	Ssal_00142	*S. thermophilus* LMG 18311 *comGA*	ABC transporter subunit	301/313 (96%)	306/313 (97%)
comGB	Ssal_00143	*S. thermophilus* LMG 18311 *comGB*	ABC transporter subunit	261/274 (95%)	268/274 (97%)
comGC	Ssal_00144	*S. thermophilus* LMD-9 *ComGC*	major pilin	107/108 (99%)	108/108 (100%)
comGD	Ssal_00145	*S. thermophilus* LMG 18311 *comGD*	minor pilin	130/142 (92%)	138/142 (97%)
comGE	Ssal_00146	*S. thermophilus ComGE*	minor pilin	68/76 (89%)	72/76 (94%)
comGF	Ssal_00147	*S. thermophilus* LMD-9 *ComGF*	minor pilin	132/145 (91%)	140/145 (96%)
comGG	Ssal_00148	*S. thermophilus comGG*	minor pilin	98/105 (93%)	103/105 (98%)

smf	Ssal_01014	*S. thermophilus* LMG 18311 *smf*	DNA processing protein, Smf family protein	265/279 (95%)	275/279 (98%)
coiA	Ssal_01737	*S. thermophilus* LMD-9 *coiA*	competence protein CoiA	269/319 (84%)	289/319 (90%)
cinA	Ssal_02112	*S. thermophilus* LMD-9 *cinA*	competence/damage-inducible protein CinA	395/423 (93%)	412/423 (97%)
ssb	Ssal_00163	*S. thermophilus* LMD-9 *ssb*	ssDNA-binding protein	128/130 (98%)	130/130 (100%)

a, – , not found. NA, No tag number was assigned.

b, Locus that shares the highest homology with is shown.

c, The length of the aligned region between the putative and target *com* genes over the length of the putative *com* gene is shown. Percent identities are shown in parentheses.

d, The length of the aligned region between the putative and target *com* genes over the length of the putative *com* gene is shown. Percent similarities are shown in parentheses.

Fig. S1.

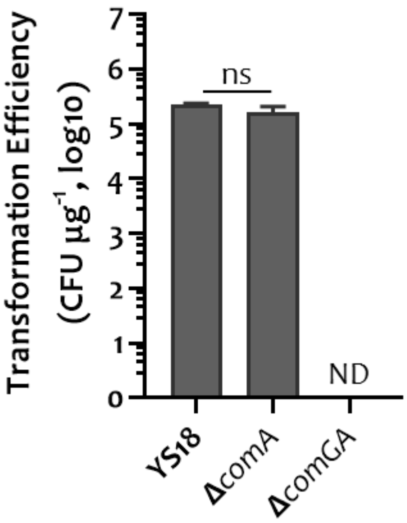

Fig. S1. Transformation efficiency of *S. salivarius* YS18 and its derivatives. Transformation efficiency is expressed as CFU µg^{-1} pDL278 in log$_{10}$. The numbers are the mean and standard deviation of three biological samples. ND, not detected. Significant differences between YS18 and its knockout derivatives were analyzed using unpaired Student's *t*-test. ns, not significant.

Fig. S2

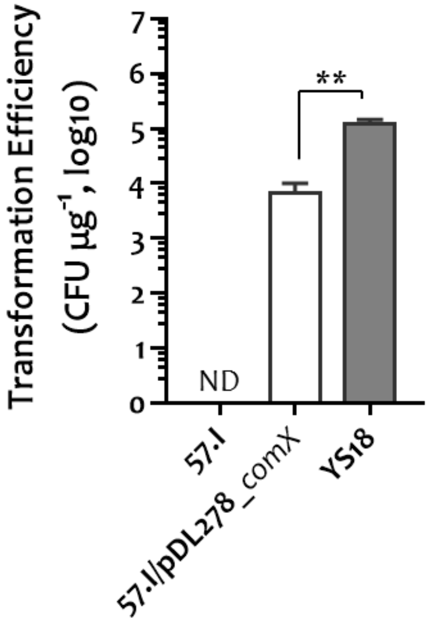

Fig. S2. Transformation efficiency of *S. salivarius* strains. Transformation efficiency is expressed as CFU µg^{-1} pDL278 in $\log_{10}$. The numbers are the mean and standard deviation of three biological samples. ND, not detected. Significant differences between YS18 and 57.I/pDL278_*comX* was analyzed using unpaired Student's *t*-test. **, *p* ,0.01.
